# Unsafe disposal of unused and expired medicines in Indonesia: nationwide evidence on public awareness and disposal practices across urban–rural and island disparities

**DOI:** 10.1186/s12889-026-27672-y

**Published:** 2026-05-18

**Authors:** Sofa D. Alfian, Meliana Griselda, Qisty A. Khoiry, Mochammad A.A Pratama, Raden M. Febriyanti, Pui San Saw, Rizky Abdulah

**Affiliations:** 1 Department of Pharmacology and Clinical Pharmacy, Faculty of Pharmacy, Universitas Padjadjaran, Jatinangor, Sumedang, West Java Indonesia; 2 Center of Excellence for Pharmaceutical Care Innovation, Universitas Padjadjaran, Jatinangor, Indonesia; 3 Department of Biology Pharmacy, Faculty of Pharmacy, Universitas Padjadjaran, Jatinangor, Indonesia; 4 School of Pharmacy, Monash University Malaysia, Subang Jaya, Selangor Darul Ehsan Malaysia

**Keywords:** Unused medicine, Expired medicine, Medication disposal practice, Awareness

## Abstract

**Background:**

Unsafe disposal of unused and expired medications is an emerging environmental and public health concern, particularly in low– and middle–income countries. In Indonesia, evidence on medication disposal practices has largely been limited to small and localized studies, constraining policy development at the national level.

**Objective:**

To assess public awareness and practices related to the disposal of unused and expired medications in Indonesia using nationally representative data, with secondary analyses stratified by residence and island to explore contextual differences.

**Methods:**

A descriptive cross–sectional study was conducted using secondary data from the 2023 Indonesian Health Survey. Respondents aged ≥ 15 years from all 38 provinces were included (*n* = 616,110). Survey–weighted descriptive analyses were performed to estimate population–level awareness of damaged medicine indicators and reported disposal practices. Analyses were additionally stratified by area of residence and island to examine contextual differences in awareness and disposal behaviors.

**Results:**

While most respondents (75.0%) recognized expiration as an indicator of damaged medicine, awareness of other critical indicators such as changes in color, odor, or packaging integrity was substantially lower. Unsafe disposal practices were widespread, with the vast majority discarding medicines in household trash (86.7%), and only a very small proportion (approximately less than 2%) returning medicines to appropriate collection systems. Stratified analyses revealed significant differences by residence and island, with rural populations more likely to engage in burning, burying, and storing medicines, while urban residents showed slightly higher awareness of non–expiration indicators. Substantial regional variation was also observed across island groups, with areas such as Maluku–Papua exhibiting higher reliance on informal disposal methods and slightly higher, yet still low, rates of medicine return.

**Conclusions:**

Public awareness of medicine deterioration beyond expiration dates remains limited, and safe disposal practices are extremely rare across Indonesia. These findings highlight critical gaps in public knowledge and access to appropriate pharmaceutical waste management systems, underscoring the urgent need for nationwide education initiatives and the development of accessible, structured medicine take–back programs. Marked differences across residence and islands indicate that medication disposal behaviors are strongly shaped by contextual and structural factors, including disparities in waste management infrastructure, healthcare access, and availability of pharmaceutical services. These findings underscore the need for geographically tailored, context–specific interventions to effectively address gaps in safe medication disposal practices across Indonesia.

**Supplementary Information:**

The online version contains supplementary material available at 10.1186/s12889-026-27672-y.

## Introduction

Unused medications, including those that are expired, deteriorated, discontinued, or no longer needed, are widely accumulated in households and represent a growing source of pharmaceutical waste worldwide [1, 2]. When improperly disposed of, such as through household trash, toilets, or washbasins, these medicines can introduce active pharmaceutical ingredients into soil and water systems, posing significant environmental and public health risks [3–6]. Unsafe medication management has been associated with medication abuse, inappropriate reuse, antimicrobial resistance, environmental contamination, and increased healthcare costs [7, 8] Empirical evidence from multiple countries demonstrates that improperly discarded antibiotics contribute to the proliferation of drug–resistant bacteria in soil [9] while pharmaceutical residues have been linked to morbidity and mortality in wildlife populations [10, 11] Despite these risks, low public awareness and limited access to safe disposal pathways continue to drive unsafe practices in many low– and middle–income settings [12–15].

In Indonesia, the challenge of unsafe disposal of pharmaceutical waste is significant. Evidence indicates that most expired and unused medicines were discarded in household trash [16]. This problem is exacerbated by the fact that the majority of Indonesians keep expired or unused medicines at home [17]. These practices are likely to vary across urban and rural settings, reflecting differences in waste management systems, pharmacy availability, and health service access [18], yet such contextual variation has rarely been examined using nationally representative data.

Studies conducted in Indonesia further suggest that medicines are frequently stored without appropriate conditions, and that visual appearance, rather than expiration status or stability considerations, is often used to judge usability, reflecting limited public awareness and the absence of structured disposal systems [19]. Such storage practices contribute not only to the accumulation of pharmaceutical waste but also to heightened risks of accidental poisoning, inappropriate reuse, and medication misuse [20].

Previous studies in Indonesia have primarily focused on selected cities or specific population groups, providing fragmented and geographically narrow insights [2, 16, 17, 19, 21, 22]. While these studies provide valuable local evidence, they are insufficient to capture national patterns across Indonesia’s diverse socio–demographic, cultural, and geographic contexts. Understanding urban–rural and island differences is particularly important in Indonesia as an archipelagic country, where disparities in healthcare access, waste management infrastructure, and health information exposure remain substantial. Evidence suggests that rural populations often have lower access to formal healthcare services and health information, while urban populations benefit from higher availability of pharmacies and structured waste systems, which may influence medication disposal behaviors and awareness levels [23–26]. These contextual differences support the use of stratified analyses to generate policy–relevant, location–specific evidence. Furthermore, comprehensive, population–level data are essential to inform national pharmaceutical waste management policies, support pharmacist–led interventions, and guide the development of scalable and sustainable medicine take–back systems. Therefore, this study aimed to assess public awareness of medicine deterioration and disposal practices of unused and expired medicines using nationally representative data from Indonesia, with secondary analyses stratified by residence and island to explore contextual differences. By simultaneously assessing awareness of medicine deterioration and reported disposal behaviors at a national scale, this study offers comprehensive evidence to inform national pharmaceutical waste management strategies.

## Methods

This study is reported in accordance with the Strengthening the Reporting of Observational Studies in Epidemiology (STROBE) [27] guidelines for cross–sectional studies (Table S1, Supplementary Data).

### Study design and data source

This study employed a descriptive cross–sectional design utilizing secondary data from the Indonesian Health Survey 2023 (IHS 2023), a nationally representative household survey conducted by the Indonesian Ministry of Health [28]. The survey employs in–person interviews and integrates multiple health modules covering communicable and non–communicable diseases, medication use, awareness and attitudes, environmental health, access to healthcare services, and sociodemographic characteristics [28]. The survey encompassed 34,500 census blocks across 38 provinces, providing nationally representative household data [28].

### Sampling and study population

The IHS 2023 applied a two–stage stratified sampling procedure. In the first stage, census blocks were proportionally selected within each regency or city by sequencing urban and rural areas according to family size. In the second stage, ordinary households and households with toddler were chosen using systematic sampling with implicit stratification based on the household head’s educational background [28]. The final dataset comprised 315,646 households and 1,191,692 individuals [28]. Inclusion criteria were respondents aged ≥ 15 years who participated in the Indonesian Health Survey 2023 and provided valid responses to medication disposal–related questions. Exclusion criteria included those with missing or incomplete responses to key outcome variables related to medication disposal awareness and practices. A complete–case analysis approach was applied, as the proportion of missing data was relatively small and the study aimed to generate descriptive population–level estimates. Given the large sample size and descriptive nature of the analysis, this approach is unlikely to introduce substantial bias.

### Variables and data collection

The analysis included variables capturing sociodemographic characteristic and Medication disposal awareness and practices. Sociodemographic characteristic including age (categorized in predefined survey age bands), gender, education level (no formal education, primary, secondary, or university), occupation, marital status (married or unmarried), island of residence (Sumatra; Java–Bali; Kalimantan; Sulawesi; Nusa Tenggara; Maluku–Papua) and area of residence. Area of residence was categorized as urban or rural based on official census classification.

Medication disposal awareness was assessed through an open–ended question asking respondents to identify characteristics indicating that medicines were damaged or no longer suitable for use across common dosage forms (tablets, syrups, ointments/creams, powders, or capsules). Responses were subsequently coded into standardized categories, including: expired medicines; damaged packaging or containers; changes in color, odor, or taste; broken or pulverized solid dosage forms; moisture, stickiness, or softening of capsules, powders, or tablets; physical changes in liquids, ointments, or creams (e.g. turbidity, precipitation, separation, or gas formation); and illegible or torn labels. To minimise misclassification bias, coding of open–ended responses followed the standardized classification framework established by the Indonesian Ministry of Health to ensure consistency across provinces and alignment with national reporting standards.

Medication disposal practices were assessed using an open–ended question on actions taken when medicines were unused, damaged, or expired. Responses were categorized into predefined options reflecting commonly reported disposal behaviors, including discarding medicines in household trash, burning or burying medicines, separating medicines from their packaging, crushing medicines before disposal, keeping medicines at home, and returning medicines to pharmacies or other authorized parties. Multiple responses were permitted, as respondents could report more than one disposal method.

All variables were self–reported and derived from standardized national survey instruments administered through face–to–face interviews by trained enumerators. Coding of open–ended responses followed the classification framework established by the Indonesian Ministry of Health to ensure consistency across provinces and alignment with national reporting standards.

### Data analysis

Descriptive statistics were used to summarize respondent characteristics and medication disposal practices. To account for the complex multi–stage sampling design, survey weights were applied across all analyses, adjusting for differential selection probabilities to ensure nationally representative estimates. Results are reported as weighted proportions with 95% confidence intervals. Awareness and disposal practice were further stratified by area of residence (urban and rural) and island group (Sumatra, Java–Bali, Kalimantan, Nusa Tenggara, Sulawesi, and Maluku–Papua). To evaluate regional disparities, chi–square tests were performed for each categorical variable across these strata, with a two–sided significance level set at p–value < 0.05. All statistical analyses were conducted using SPSS version 27.0 (IBM Corp., New York, USA).

## Results

### Respondents’ characteristics

**Fig. 1:**
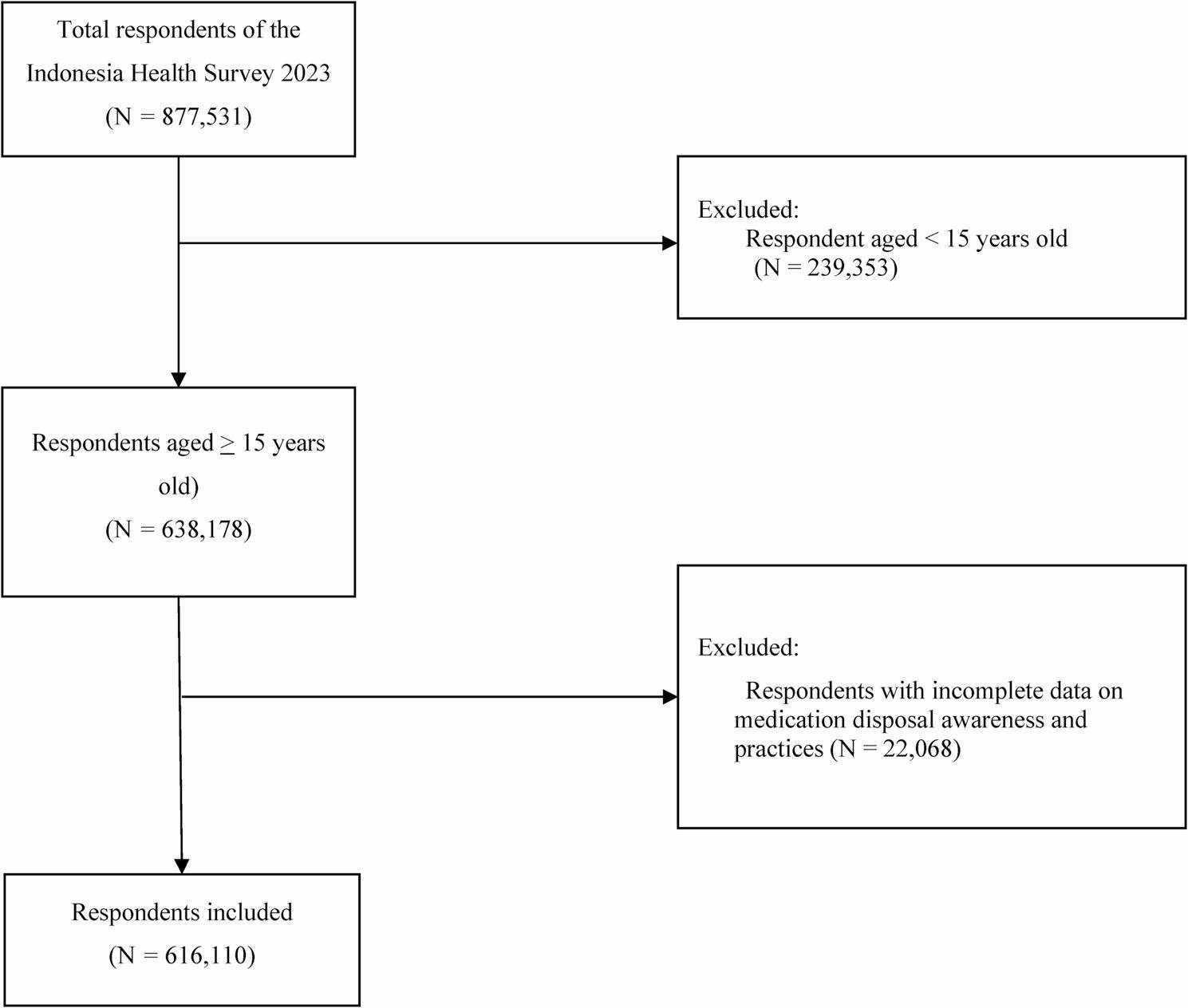
Respondent selection process

Among the 877,531 individuals participated in the survey, 239,353 individuals aged < 15 years were excluded (Fig. 1). Among the remaining 638,178 eligible respondents, 22,068 individuals were excluded due to missing data on medication disposal awareness or practices. The final analytical sample therefore comprised 616,110 respondents, of whom 58.7% resided in urban areas and 41.3% in rural areas.

Table 1 presents the overall and urban–rural stratified sociodemographic characteristics. The largest age group was 35–44 years (20.3%), followed by 25–34 years (21.6%). In the unweighted sample, females constituted a slight majority of respondents (54.6%). However, after applying survey weights to obtain nationally representative estimates, females accounted for approximately half of the population (49.9%). Most respondents had completed secondary education, with high school as the highest level attained by 33.3% of participants. More than one–third of respondents were unemployed (37.6%), and the majority were married (69.4%). Over half resided in urban areas (58.7%). In terms of geographic distribution, respondents were predominantly from Java–Bali (58.6%), followed by Sumatra (21.2%), with smaller proportions from other island groups (Table 1).

**Table 1:** Sociodemographic characteristics of respondents overall and by urban–rural residence

Characteristic	Rural (*N* = 285,556)	Urban (*N* = 330,554)	*p*–value	Total (*N* = 616,110)
	% (95% CI) Weighted	% (95% CI) Weighted		% (95% CI) Weighted
Age, in years			< 0.001*	
15–24	19.65 (19.33–19.97)	19.06 (18.81–19.3)		19.30 (19.10–19.50)
25–34	21.64 (21.32–21.95)	21.52 (21.24–21.8)		21.57 (21.36 − 21.78)
35–44	19.98 (19.7–20.25)	20.54 (20.29–20.79)		20.31 (20.12–20.49)
45–54	17.21 (16.98–17.45)	17.73 (17.51–17.94)		17.51 (17.36–17.67)
55–64	12.47 (12.26–12.69)	12.57 (12.39–12.76)		12.53 (12.39–12.67)
* ≥*65	9.05 (8.85–9.26)	8.59 (8.41–8.78)		8.78 (8.65–8.92)
Gender			0.054	
Female	50.34 (50.09–50.59)	50.01 (49.78–50.23)		49.86 (49.69–50.02)
Male	49.66 (49.41–49.91)	49.99 (49.77–50.22)		50.14 (49.98–50.31)
Education level			< 0.001^*^	
Did not attend school	14.81 (14.41–15.21)	7.28 (7.08–7.5)		10.39 (10.18–10.61)
Elementary school	33 (32.49–33.52)	19.59 (19.19–19.99)		25.13 (24.79–25.48)
Middle school	22.57 (22.24–22.91)	20.37 (20.1–20.65)		21.28 (21.07–21.5)
High school	24.1 (23.7–24.5)	39.8 (39.35–40.25)		33.31 (32.96–33.66)
University	5.52 (5.35–5.69)	12.96 (12.64–13.27)		9.88 (9.68–10.09)
Occupation			< 0.001^*^	
Unemployed	35.57 (35.15–35.99)	38.95 (38.64–39.26)		37.56 (37.30–37.81)
Government/private sector	7.29 (7.05–7.55)	20.31 (19.91–20.71)		17.33 (17.01–17.65)
Self–employed/entrepreneur	13.28 (12.94–13.62)	17.46 (17.17–17.76)		15.73 (15.51–15.96)
Farmer/fisherman	31.62 (31.1–32.13)	7.27 (7.04–7.5)		14.93 (14.65–15.22)
Laborer/driver/household assistant	5.79 (5.49–6.11)	10.08 (9.81–10.37)		8.31 (8.10–8.52)
Other	6.45 (6.22–6.7)	5.93 (5.75–6.1)		6.14 (6.00–6.29)
Marital status			< 0.001^*^	
Unmarried	28.49 (28.13–28.85)	32.05 (31.74–32.37)		30.58 (30.34–30.82)
Married	71.51 (71.15–71.87)	67.95 (67.63–68.26)		69.42 (69.18–69.66)
Island of residence			< 0.001^*^	
Sumatera	27.46 (26.5–28.45)	16.76 (16.07–17.48)		21.18 (20.61–21.77)
Java–Bali	45.06 (43.7–46.43)	68.1 (67.18–69.00)		58.58 (57.80–59.35)
Sulawesi	9.94 (9.48–10.41)	5.4 (5.07–5.75)		7.28 (7.01–7.56)
Kalimantan	7.27 (6.86–7.71)	5.28 (4.97–5.62)		6.11 (5.85–6.37)
Maluku– Papua	4.86 (4.5–5.25)	1.86 (1.68–2.06)		3.1 (2.92–3.30)
Nusa Tenggara	5.4 (4.98–5.85)	2.6 (2.37–2.84)		3.76 (3.53–3.99)

*Statistically significant (*p* < 0.05)

**Table 2:** Sociodemographic characteristics of respondents by island

Characteristic	% Weighted (95% CI)						*p*–value
	Sumatera	Java and Bali	Kalimantan	Nusa Tenggara	Sulawesi	Maluku and Papua	
Gender							< 0.001^*^
Male	50.2 (50.0–50.5)	50 (49.7–50.2)	51.2 (50.8–51.7)	49.5 (48.9–50.1)	50 (49.5–50.4)	52 (51.0–52.9)	
Female	49.8 (49.5–50.0)	50 (49.8–50.3)	48.8 (48.3–49.2)	50.5 (49.9–51.1)	50 (49.6–50.5)	48 (47.1–49.0)	
Marital Status							< 0.001^*^
Unmarried	31.1 (30.7–31.5)	30.3 (30.0–30.7)	31 (30.4–31.7)	30.9 (29.9–31.9)	32 (31.5–32.5)	27.4 (26.3–28.5)	
Married	68.9 (68.5–69.3)	69.7 (69.3–70.0)	69 (68.3–69.6)	69.1 (68.1–70.1)	68 (67.5–68.5)	72.6 (71.5–73.7)	
Age, in years							< 0.001^*^
15–24	20.4 (20.1–20.8)	18.3 (18.0–18.6)	20.5 (19.9–21.0)	21.4 (20.6–22.1)	21.1 (20.6–21.5)	21.3 (20.2–22.4)	
25–34	22.5 (22.2–22.9)	20.7 (20.4–21.0)	22.8 (22.3–23.3)	23.3 (22.6–24.1)	22.4 (21.9–22.8)	24.7 (23.7–25.7)	
35–44	20.6 (20.3–20.9)	20.1 (19.8–20.4)	21.1 (20.7–21.6)	20.3 (19.6–20.9)	19.7 (19.3–20.1)	21.9 (20.8–22.9)	
45–54	16.9 (16.7–17.2)	18 (17.7–18.2)	17.3 (16.9–17.7)	16.3 (15.8–16.8)	16.6 (16.3–16.9)	16.6 (15.7–17.6)	
55–64	11.6 (11.4–11.8)	13.3 (13.1–13.6)	11.3 (10.9–11.6)	11 (10.5–11.5)	11.7 (11.4–12.0)	10 (9.3–10.6)	
*≥* 65	7.9 (7.7–8.1)	9.6 (9.3–9.8)	7 (6.7–7.4)	7.8 (7.4–8.2)	8.5 (8.2–8.8)	5.6 (5.1–6.1)	
Occupation							< 0.001^*^
Unemployed	39.6 (39.2–40.0)	36.3 (36.0–36.7)	38.5 (37.9–39.1)	33.7 (32.9–34.6)	41.7 (41.1–42.3)	39.4 (37.9–41.0)	
Government/private sector	9.5 (9.2–9.9)	17.8 (17.3–18.2)	17 (16.3–17.7)	10.3 (9.7–11.0)	9.8 (9.4–10.3)	11.8 (10.7–12.9)	
Self–employed/entrepreneur	16 (15.6–16.5)	16.9 (16.6–17.2)	14.8 (14.3–15.4)	10.7 (10.0–11.4)	11.8 (11.4–12.3)	9 (8.2–9.9)	
Farmer/fisherman	22.3 (21.7–22.9)	13.5 (13.0–13.9)	18.2 (17.4–19.0)	32 (30.7–33.3)	20.7 (20.1–21.4)	29.1 (27.3–31.1)	
Laborer/driver/household assistant	5.3 (5.0–5.5)	10.9 (10.6–11.2)	4.6 (4.3–4.9)	4.6 (4.1–5.2)	4 (3.7–4.3)	2.2 (1.9–2.4)	
Other	7.3 (7.0–7.6)	4.6 (4.4–4.8)	6.9 (6.5–7.3)	8.7 (8.1–9.3)	11.9 (11.5–12.4)	8.5 (7.9–9.2)	
Area of residence							< 0.001^*^
Urban	46.4 (45.0–47.8)	68.2 (66.9–69.5)	50.8 (48.7–52.8)	40.6 (37.7–43.5)	43.6 (41.7–45.4)	35.2 (32.3–38.1)	
Rural	53.6 (52.2–55.0)	31.8 (30.5–33.1)	49.2 (47.2–51.3)	59.4 (56.5–62.3)	56.4 (54.6–58.3)	64.8 (61.9–67.7)	
Education							< 0.001^*^
Did not attend school	9.4 (9.1–9.7)	9.5 (9.2–9.9)	11.5 (11.0–12.0)	16.9 (15.9–18.0)	10.7 (10.3–11.2)	22.2 (20.2–24.4)	
Elementary school	21.4 (20.9–21.8)	27.4 (26.8–27.9)	25.7 (24.9–26.5)	23.5 (22.5–24.4)	23 (22.4–23.6)	14.2 (13.4–15.0)	
Middle school	22.3 (22.0–22.7)	21.5 (21.1–21.8)	21 (20.5–21.5)	18.6 (17.9–19.3)	20 (19.6–20.4)	17.5 (16.6–18.3)	
High school	37.1 (36.6–37.7)	32.3 (31.8–32.9)	31.5 (30.8–32.3)	28.6 (27.7–29.5)	33.8 (33.1–34.4)	33.9 (32.2–35.6)	
University	9.7 (9.4–10.0)	9.3 (9.0–9.6)	10.3 (9.8–10.9)	12.5 (11.8–13.2)	12.5 (12.0–13.1)	12.2 (11.0–13.5)	

*Statistically significant (*p* < 0.05)

### Awareness of damaged medicine indicators

Table 3 presents awareness overall and stratified by urban and rural residence. Overall, awareness of indicators of damaged medicines varied substantially across response categories. The most frequently reported indicator was expiration, identified by 73.3% of respondents. Other indicators were less commonly recognized, including damaged packaging or containers (42.0%), changes in color, odor, or taste (35.6%), and broken or pulverized solid dosage forms (32.1%). Recognition of more subtle signs of deterioration was comparatively low, with moisture or stickiness of capsules, powders, or tablets reported by 20.5% of respondents, physical changes in liquids, ointments, or creams by 15.7%, and illegible or torn labels by 14.9%.

**Table 3:** Respondents’ awareness of damaged medicine signs and disposal practices by residency

Questions	Responses	Rural (*N* = 285,556)	Urban (*N* = 330,554)	p–value	Total (*N* = 616,110)
		% (95% CI) Weighted	% (95% CI) Weighted		% (95% CI) Weighted
What are the characteristics of damaged medicines in the form of tablets, syrups, ointments/creams, powders, or capsules?	Expired medicines	71.75 (71.19–72.3)	74.31 (73.85–74.77)	< 0.001^*^	73.25 (72.90–73.61)
	Damaged container/packaging	40.2 (39.55–40.85)	43.18 (42.67–43.69)	< 0.001^*^	41.95 (41.54–42.35)
	Changes in color, odor, or taste	33.11 (32.47–33.76)	37.26 (36.75–37.77)	< 0.001^*^	35.55 (35.14–35.95)
	Broken, cracked, perforated, or pulverized medicines	33.1 (32.45–33.75)	31.41 (30.92–31.92)	< 0.001^*^	32.11 (31.71–32.51)
	Capsules, powders, or tablets that were moist, softened, wet, or sticky	20.72 (20.19–21.26)	20.27 (19.84–20.71)	0.206	20.46 (20.12–20.80)
	Liquids, ointments, or creams that were turbid, thickened, precipitated, separated, hardened, or showed stains, spots, or gas formation	16.19 (15.69–16.7)	15.33 (14.94–15.74)	0.009^*^	15.69 (15.38–16.01)
	Illegible or torn labels	15.21 (14.73–15.7)	14.61 (14.21–15.02)	0.060	14.86 (14.55–15.17)
What did you do with medicines that can no longer be used, damaged, or expired?	Discarded in the trash	85.49 (85.07–85.89)	87.46 (87.15–87.76)	< 0.001^*^	86.64 (86.39–86.89)
	Burning or burying medicines	18.24 (17.72–18.77)	10.55 (10.23–10.87)	< 0.001^*^	13.72 (13.43–14.02)
	Separating medicines from their packaging	8.28 (7.92–8.66)	8.9 (8.61–9.2)	0.011^*^	8.64 (8.42–8.88)
	Keeping them at home	10.14 (9.79–10.51)	6.83 (6.62–7.05)	< 0.001^*^	8.20 (8.01–8.40)
	Crushing medicines before disposal	3.99 (3.77–4.24)	5.89 (5.66–6.14)	< 0.001^*^	5.11 (4.94–5.28)
	Returning to pharmacies or third parties	1.64 (1.55–1.73)	1.53 (1.45–1.62)	0.088	1.57 (1.51–1.64)

*Statistically significant (*p* < 0.05)

Urban respondents consistently demonstrated higher recognition of damaged packaging (43.2% vs. 40.2%, *p* < 0.001) and changes in color, odor, or taste (37.3% vs. 33.1%, *p* < 0.001) compared with rural respondents. Recognition of expiration as a damage indicator was similarly high in both settings and was slightly higher among urban than rural residents (74.3% vs. 71.8%, *p* < 0.001) (Table 3). Notably, while recognition of expiration dates was high (> 70%), the majority of respondents failed to identify other critical indicators of medicine deterioration.

Awareness levels stratified by island group are presented in Table 4, with statistically significant differences observed across all indicators (all *p* < 0.001). Recognition of expiration dates was most prevalent in Kalimantan (77.9%) and Sumatra (77.2%), while Nusa Tenggara reported the lowest awareness at 68.5%. For damaged packaging, awareness ranged from a high of 46.3% in Maluku–Papua to a low of 33.7% in Nusa Tenggara. Similarly, recognition of changes in color, odor, or taste peaked in Maluku–Papua (43.1%) and Sumatra (39.4%), contrasting with the lower levels found in Sulawesi (33.4%) and Java–Bali (33.8%). Notably, despite having the most rural population profile, Maluku–Papua consistently outperformed other regions in identifying non–expiration deterioration indicators, including broken solid dosage forms (44.7%), physical changes in liquids/creams (28.6%), and illegible labels (25.2%).

**Table 4:** Respondents’ awareness of damaged medicine signs by island

Characteristic	% Weighted (95% CI)							*p*–value
	Sumatera	Javav Bali	Kalimantan	Nusa Tenggara	Sulawesi	Malukuv Papua		
Broken, cracked, perforated, or pulverized medicines								
Yes	39.5 (38.7–40.3)	28.1 (27.5–28.6)	38.3 (37.1–39.5)	32.4 (31.0–33.8)	32.4 (31.5–33.3)		44.7 (42.5–46.9)	< 0.001^*^
No	60.5 (59.7–61.3)	71.9 (71.4–72.5)	61.7 (60.5–62.9)	67.6 (66.2–69.0)	67.6 (66.7–68.5)		55.3 (53.1–57.5)	
Changes in color, odor, or taste								
Yes	39.4 (38.6–40.2)	33.8 (33.3–34.4)	37.1 (35.9–38.3)	35.7 (34.3–37.1)	33.4 (32.5–34.3)		43.1 (40.7–45.5)	< 0.001^*^
No	60.6 (59.8–61.4)	66.2 (65.6–66.7)	62.9 (61.7–64.1)	64.3 (62.9–65.7)	66.6 (65.7–67.5)		56.9 (54.5–59.3)	
Damaged container/packagin**g**								
Yes	44.6 (43.8–45.3)	41.7 (41.1–42.3)	44.6 (43.4–45.8)	33.7 (32.2–35.1)	36.7 (35.8–37.6)		46.3 (44.0–48.7)	< 0.001^*^
No	55.4 (54.7–56.2)	58.3 (57.7–58.9)	55.4 (54.2–56.6)	66.3 (64.9–67.8)	63.3 (62.4–64.2)		53.7 (51.3–56.0)	
Liquids, ointments, or creams that were turbid, thickened, precipitated, separated, hardened, or showed stains, spots, or gas formation								
Yes	20 (19.4–20.7)	13 (12.6–13.4)	19.8 (18.8–20.8)	16.3 (15.2–17.4)	15.3 (14.5–16.0)		28.6 (26.1–31.3)	< 0.001^*^
No	80 (79.3–80.6)	87 (86.6–87.4)	80.2 (79.2–81.2)	83.7 (82.6–84.8)	84.7 (84.0–85.5)		71.4 (68.7–73.9)	
Capsules, powders, or tablets that were moist, softened, wet, or sticky								
Yes	24.2 (23.5–24.9)	18 (17.6–18.5)	25.9 (24.8–27.1)	18.7 (17.6–19.8)	19.9 (19.1–20.8)		33.5 (31.0–36.0)	< 0.001^*^
No	75.8 (75.1–76.5)	82 (81.5–82.4)	74.1 (72.9–75.2)	81.3 (80.2–82.4)	80.1 (79.2–80.9)		66.5 (64.0–69.0)	
Illegible or torn labels								
Yes	18.5 (17.9–19.1)	12.7 (12.3–13.1)	17.8 (16.8–18.9)	15.1 (14.0–16.3)	14.7 (13.9–15.5)		25.2 (22.8–27.9)	< 0.001^*^
No	81.5 (80.9–82.1)	87.3 (86.9–87.7)	82.2 (81.1–83.2)	84.9 (83.7–86.0)	85.3 (84.5–86.1)		74.8 (72.1–77.2)	
Expired medicines								
Yes	77.2 (76.7–77.8)	71.3 (70.7–71.8)	77.9 (77.0–78.8)	68.5 (67.1–69.9)	76.9 (76.2–77.6)		71.8 (70.0–73.6)	< 0.001^*^
No	22.8 (22.2–23.3)	28.7 (28.2–29.3)	22.1 (21.2–23.0)	31.5 (30.1–32.9)	23.1 (22.4–23.8)		28.2 (26.4–30.0)	

*Statistically significant (*p* < 0.05)

### Medication disposal practices

Table 3 presents disposal practices overall and stratified by urban and rural residence. The predominant disposal method was discarding medicines in household trash, reported by 86.6% of respondents. This behavior was more prevalent in urban areas (87.5%) compared with rural areas (85.5%, *p* < 0.001). Burning or burying medicines was significantly more common in rural areas (18.2% vs. 10.6%, *p* < 0.001) and crushing before disposal (5.9% vs. 3.9%, *p* < 0.001). Separating medicines from packaging before disposal was significantly more frequent in urban areas (8.9% vs. 8.6%, *p* = 0.011), though the absolute difference was minimal. Only 1.6% of urban and 1.5% of rural respondents reported returning medicines to pharmacies or other authorized parties. This difference was not statistically significant (*p* = 0.088); accordingly, the rate of medicine return to pharmacies was comparable between urban and rural settings.

Disposal practices stratified by island group are detailed in Table 5, revealing statistically significant inter–regional differences across all indicators (all *p* < 0.001). Disposal via household trash remained the predominant method nationwide, peaking at 88.7% in Kalimantan and reaching its lowest point in Maluku–Papua (78.0%). In contrast, burning or burying was most prevalent in Maluku–Papua (23.7%), while Sulawesi reported the lowest frequency at 10.8%. Maluku–Papua (20.3%) and Nusa Tenggara (13.3%) also showed the highest rates of keeping unused medicines at home, a practice significantly less common in Kalimantan (6.5%) and Java–Bali (6.7%). Notably, returning medicines to authorized parties remained extremely rare regardless of geography; even the highest rate observed in Maluku–Papua was only 3.9%, compared to a low of 1.2% in Java–Bali. Despite these regional variations, the absolute rates of safe disposal remain critically low across all island groups without exception.

**Table 5:** Respondents’ disposal practices by island

Characteristics	% Weighted (95% CI)						*p*–value
	Sumatera	Javaz– Bali	Kalimantan	Nusa Tenggara	Sulawesi	Malukuv– Papua	
Separating medicines from their packaging							
Yes	10.7 (10.2–11.1)	7.2 (6.9–7.6)	10.5 (9.9–11.3)	7.9 (7.2–8.7)	9.5 (9.0–10.1)	16.2 (14.7–17.9)	< 0.001^*^
No	89.3 (88.9–89.8)	92.8 (92.4–93.1)	89.5 (88.7–90.1)	92.1 (91.3–92.8)	90.5 (89.9–91.0)	83.8 (82.1–85.3)	
Crushing medicines before disposal							
Yes	5.1 (4.9–5.4)	5 (4.8–5.3)	4.5 (4.2–4.9)	4.7 (4.2–5.3)	4.4 (4.0–4.8)	9.6 (8.4–10.9)	< 0.001^*^
No	94.9 (94.6–95.1)	95 (94.7–95.2)	95.5 (95.1–95.8)	95.3 (94.7–95.8)	95.6 (95.2–96.0)	90.4 (89.1–91.6)	
Keeping them at home							
Yes	9.6 (9.2–10.0)	6.7 (6.4–6.9)	6.5 (6.0–6.9)	13.3 (12.4–14.3)	10.2 (9.7–10.7)	20.3 (18.4–22.2)	< 0.001^*^
No	90.4 (90.0–90.8)	93.3 (93.1–93.6)	93.5 (93.1–94.0)	86.7 (85.7–87.6)	89.8 (89.3–90.3)	79.7 (77.8–81.6)	
Discarded in the trash							
Yes	87.5 (87.0–87.9)	86.6 (86.2–86.9)	88.7 (88.1–89.4)	83.9 (82.7–85.0)	88.1 (87.6–88.6)	78 (75.9–80.0)	< 0.001*
No	12.5 (12.1–13.0)	13.4 (13.1–13.8)	11.3 (10.6–11.9)	16.1 (15.0–17.3)	11.9 (11.4–12.4)	22 (20.0–24.1)	
Burning or burying medicines							
Yes	15.9 (15.3–16.5)	12.6 (12.2–13.0)	15.1 (14.2–16.0)	14.7 (13.7–15.8)	10.8 (10.3–11.4)	23.7 (21.5–26.1)	< 0.001^*^
No	84.1 (83.5–84.7)	87.4 (87.0–87.8)	84.9 (84.0–85.8)	85.3 (84.2–86.3)	89.2 (88.6–89.7)	76.3 (73.9–78.5)	
Returning to pharmacies or third parties							
Yes	1.9 (1.8–2.0)	1.2 (1.2–1.3)	1.7 (1.5–1.8)	2.1 (1.8–2.3)	2 (1.8–2.3)	3.9 (3.2–4.7)	< 0.001^*^
No	98.1 (98.0–98.2)	98.8 (98.7–98.8)	98.3 (98.2–98.5)	97.9 (97.7–98.2)	98 (97.7–98.2)	96.1 (95.3–96.8)	

*Statistically significant (*p* < 0.05)

## Discussion

This nationwide analysis provides the most comprehensive evidence to date on public awareness and disposal practices of unused and expired medicines in Indonesia. Importantly, the urban–rural stratified analysis reveals systematic contextual differences that are masked in national‑level aggregates. The most critical finding of this nationwide analysis is not the disparity between urban and rural settings, but rather the near–universal absence of safe disposal practices across all geographic strata. The prop

ortion of respondents returning unused or expired medicines to pharmacies or authorized collection facilities remained below 2% in all residence categories and under 4% across all island groups. These figures underline a near–complete failure of pharmaceutical take–back systems to reach the Indonesian public at scale. Consequently, this systemic gap in safe disposal channels must be prioritized as a critical public health concern, as the current infrastructure is virtually non–existent in practice regardless of geography [29].

Urban–rural stratified analyses revealed several statistically significant differences in disposal behavior. Rural residents were significantly more likely to burn or bury medicines and to keep them at home, a trend that likely reflects limited formal waste collection services and fewer accessible pharmacy–based disposal options. Conversely, urban residents more frequently disposed of medicines in household trash while demonstrating significantly higher awareness of non–expiration damage indicators. However, the most policy–relevant urban–rural comparison, namely the rate of returning medicines to pharmacies, was not statistically significant. This parity indicates that urban settings provide no meaningful advantage in driving safe pharmaceutical disposal despite better infrastructure. Ultimately, these patterns confirm that unsafe disposal is not primarily an individual knowledge deficit but is instead deeply shaped by structural and infrastructural constraints that persist even in urban environments [30], including the absence of organized systems and integrated policy frameworks [31].

Beyond this urban–rural divide, substantial geographic heterogeneity was observed across the six major island groups, with Maluku–Papua displaying the most distinct disposal profile. This region reported the highest rates of burning or burying (23.7%), keeping medicines at home (20.3%), and returning them to pharmacies (3.9%). These patterns likely reflect limited access to formal municipal waste management and healthcare infrastructure, necessitating a reliance on burning or stockpiling as de facto disposal strategies [32]. Conversely, the higher pharmacy return rates in this area may suggest a deeper community reliance on pharmacies as primary healthcare touchpoints in underserved regions [33, 34].

Similar trends of stockpiling were noted in Nusa Tenggara, which is consistent with its rural and agricultural demographic. Meanwhile, Kalimantan and Sulawesi exhibited the highest rates of household trash disposal. Although this is the most common method nationally, it remains an environmentally unsafe practice that leads to pharmaceutical contamination of soil and water systems. Perhaps most strikingly, Java–Bali, despite being the most urbanized region with the most robust healthcare infrastructure, reported the lowest rate of pharmacy returns. This paradox highlights that the mere physical proximity of pharmacies does not ensure their use as disposal points in the absence of structured and publicly promoted take–back programs. Ultimately, these findings provide a roadmap for the Government of Indonesia to design regionally tailored pharmaceutical waste policies and targeted nationwide campaigns.

Beyond these geographic disparities, the study also reveals a significant knowledge gap regarding medicine quality. Although most respondents correctly identified expiration as an indicator of damaged medicines, substantially fewer recognized other clinically and pharmaceutically important signs, such as changes in physical appearance, compromised packaging, or labeling issues. This pattern suggests that public understanding of medicine quality is narrowly focused on expiration dates, overlooking other critical indicators of deterioration. Such limited awareness may delay appropriate disposal and contribute to the prolonged storage of unusable medicines in households, thereby increasing the risk of inappropriate reuse, accidental ingestion, and misuse [20]. These findings highlight a critical gap in public knowledge that represents a key barrier to safe medication management. Similar findings have been reported in previous city–level studies in Indonesia [2, 16], this suggests that low awareness is a systemic issue rather than a localized phenomenon. Furthermore, urban residents often demonstrate higher levels of health literacy and greater access to health information and health services compared with rural residents, which may contribute to higher awareness of non–expiration medicine deterioration indicators [23, 24]. Conversely, rural residents were more likely to engage in burning or burying medicines, practices that may be driven by limited formal waste collection services and fewer accessible pharmacy‑based disposal options. Furthermore, the higher tendency to retain unused medicines in rural households may further reflect perceived future need, geographic barriers to healthcare access, and lower perceived environmental risk. These patterns highlight that unsafe disposal is not solely an individual knowledge issue but is strongly shaped by structural and infrastructural constraints [25, 26].

We observed that unsafe disposal practices were widespread, with most respondents discarding unused or expired medicines in the household trash. This finding aligns with studies from Indonesia [16] and other low– and middle–income countries, including Afghanistan [35], Ethiopia [36], Serbia [37], Malaysia [38], India [39], and Saudi Arabia [40], where discarding medicines in household waste is the dominant disposal method. Convenience, low perceived environmental or health risk, and the absence of accessible disposal infrastructure likely contribute to the persistence of this behavior [41]. However, pharmaceutical residues originating from landfill waste and sewage systems have been repeatedly detected in soil, surface water, and groundwater, where they have been associated with antimicrobial resistance, endocrine disruption, ecological toxicity, and potential human health impacts [42–44]. The continued reliance on household trash disposal therefore highlights the urgent need for population–wide education and strengthened pharmaceutical waste management systems.

Burning or burying medicines emerged as the second most frequently reported disposal method. While less prevalent than disposal in household trash, this practice similarly reflects limited access to regulated disposal pathways [2, 7]. Comparable behaviors have been documented in Ethiopia [36] and Serbia [37], particularly in rural areas where formal waste management systems are limited. Although burning or burying medicines may be perceived as safer alternatives to direct disposal in trash, neither method aligns with environmentally sound pharmaceutical waste management principles. International guidelines continue to emphasize high–temperature incineration through regulated collection systems as the recommended approach for minimizing environmental contamination [35, 45].

We further observed that a proportion of respondents reported separating medicines from their packaging and crushing medicines prior to disposal. Although these practices are not equivalent to safe disposal, they may represent attempts to minimize risks associated with direct disposal, such as preventing misuse, limiting scavenging, or preventing access by children. Similar precautionary behaviors have been reported in Indonesia, Ethiopia, and Nigeria, where crushing medicines before disposal is commonly practiced once medicines enter household waste [14, 36, 46–48]. The separation of medicines from their packaging, though less frequently described in the literature, may likewise represent an effort to render medicines less recognizable or retrievable in the absence of formal guidance [18]. Additionally, the tendency to retain unused or expired medicines at home, observed in this study, has been attributed in previous research to limited awareness of appropriate disposal methods [2, 14] or to perceived future need [49], a behavior also documented in Ireland Ireland [49] and Afghanistan [35]. Collectively, these findings illustrate how individuals may adopt improvised disposal strategies in settings where structured take–back systems are unavailable.

Of particular concern is the extremely low proportion of respondents who reported returning unused or expired medicines to pharmacies or authorized collection systems, indicating a critical gap in access to safe disposal infrastructure. This finding suggests that improper disposal is not merely a behavioral issue but reflects systemic limitations in pharmaceutical waste management and insufficient public awareness of available disposal options. In contrast, countries such as South Korea [50], Sweden [51], Australia [52], France [53], Belgium, Italy, Greece, and Norway [54], have long–standing, well–publicized pharmacy–based take–back systems that are embedded within routine pharmaceutical care. Indonesia’s initiatives remain limited, inconsistent, or poorly promoted [16, 55, 56]. In Indonesia, although a national medication take–back initiative (“Let’s Dispose of Medication Waste Properly”) was launched in 2019 by the Indonesian Food and Drug Authority [57], its implementation has not been sustained at scale and public participation remains extremely low. These findings are consistent with recent surveys among Indonesian pharmacists, which identified limited training, unclear operational guidance, and low public awareness as key barriers to effective take–back program implementation [56, 58, 59] The consistently low prevalence of medicine return to pharmacies across both urban and rural settings underscores systemic gaps in Indonesia’s pharmaceutical waste management framework. While urban settings may be better positioned to implement pharmacy‑based take‑back programs, tailored community‑based collection strategies may be required in rural areas to ensure equitable access.

These results have important implications for public health policy and practice. First, structured and sustainable pharmacy–based take–back programs are urgently needed, supported by clear national guidelines, logistics systems, and reporting mechanisms. Such programs must be supported by clear national guidelines, logistics systems, and formal reporting mechanisms to move beyond the current fragmented initiatives. Second, routine public education campaign, integrated into primary care services, community health centers, pharmacies, and digital media, should emphasize not only the environmental and health risks of unsafe disposal but also the full spectrum of indicators of medicine deterioration. Third, the role of pharmacists should be strengthened, as international evidence consistently shows that pharmacist–led interventions effectively reduce household pharmaceutical waste and improve disposal practices. Expanding professional training and incorporating pharmaceutical waste management into pharmacy curricula may further support long–term behavior change [56, 59]. However, interventions focusing solely on public education are unlikely to be sufficient without parallel investment in accessible disposal infrastructure and institutionalized pharmacy–based take–back systems.

The strength of this study is the large, nationally representative sample, which provides the most comprehensive evidence to date on medication disposal practices in Indonesia. The application of survey weighting enhances the generalizability of the findings across diverse demographic and geographic contexts. Nevertheless, several limitations warrant consideration. First, the reliance on self–reported data may introduce recall or social desirability bias. Second, medication disposal practices were assessed using open–ended questions that were subsequently categorized, which may have introduced misclassification bias despite standardized coding procedures. Third, the survey did not capture the frequency or consistency of disposal behaviors, limiting differentiation between habitual and occasional practices. Fourth, the absence of information on medicine type, quantity, or therapeutic class limits assessment of the environmental risk burden. Fifth, the survey does not capture contextual factors such as local availability of take–back programs, pharmacy counseling exposure, or waste management infrastructure. Future research should therefore move toward analytic study designs, such as multivariable regression, to identify specific individual–level predictors of unsafe disposal. This approach should incorporate subgroup analyses, objective behavioral measures, and longitudinal evaluations of pharmacy–based take–back interventions. Such evidence will be pivotal in informing targeted behavior change strategies and supporting the development of robust, sustainable, and evidence–based pharmaceutical waste management policies across Indonesia.

## Conclusion

Public awareness of medicine deterioration beyond expiration dates remains limited, and safe disposal practices are extremely rare across Indonesia. These findings highlight critical gaps in public knowledge and access to appropriate pharmaceutical waste management systems, underscoring the urgent need for nationwide education initiatives and the development of accessible, structured medicine take–back programs. Distinct urban–rural differences indicate that disposal behaviors are context‑dependent, reinforcing the need for differentiated policy responses, including pharmacy‑based take‑back systems in urban areas and community‑integrated disposal solutions in rural settings. Significant differences across island groups further reveal that disposal behaviors are shaped by geographic and infrastructural context, reinforcing the need for regionally differentiated policy responses tailored to Indonesia’s diverse archipelagic landscape.

## Supplementary Information

Supplementary Material 1.

## Data Availability

The dataset analysed during the current study is publicly available from the Indonesian Ministry of Health through the Indonesian Health Survey (IHS) 2023 repository.

## References

[1] Law AV, Sakharkar P, Zargarzadeh A, Tai BWB, Hess K, Hata M, et al. Taking stock of medication wastage: Unused medications in US households. Res Social Administrative Pharm. 2015;11:571–8. 10.1016/j.sapharm.2014.10.003. 10.1016/j.sapharm.2014.10.003 25487420

[2] Alfian SD, Insani WN, Halimah E, Qonita NA, Jannah SS, Nuraliyah NM, et al. Lack of Awareness of the Impact of Improperly Disposed Of Medications and Associated Factors: A Cross–Sectional Survey in Indonesian Households. Front Pharmacol. 2021;12. 10.3389/fphar.2021.630434. 10.3389/fphar.2021.630434 PMC8107817 33981221

[3] Daughton CG, Pharmaceuticals, the Environment (PiE). Evolution and impact of the published literature revealed by bibliometric analysis. Sci Total Environ. 2016;562:391–426. 10.1016/j.scitotenv.2016.03.109. 27104492 10.1016/j.scitotenv.2016.03.109

[4] Azmi Hassali M, Shakeel S. Unused and Expired Medications Disposal Practices among the General Public in Selangor, Malaysia. Pharmacy. 2020;8:196. 10.3390/pharmacy8040196. 33114172 10.3390/pharmacy8040196 PMC7712208

[5] Kassahun H, Tesfaye D. Disposal Practices of Unused Medications Among Patients in Public Health Centers of Dessie Town, Northeast Ethiopia: A Cross–sectional Study. Curr Drug Saf. 2020;15:105–10. 10.2174/1574886315666200331140400. 32228428 10.2174/1574886315666200331140400

[6] Marwa KJ, Mcharo G, Mwita S, Katabalo D, Ruganuza D, Kapesa A. Disposal practices of expired and unused medications among households in Mwanza, Tanzania. PLoS ONE. 2021;16:e0246418. 10.1371/journal.pone.0246418. 33539402 10.1371/journal.pone.0246418 PMC7861449

[7] Koshok M, Jan T, ALtawil S, Alghamdi E, Ali A, Sobh A, et al. Awareness of home drug storage and utilization habits: Saudi study. Med Sci | Int Med J. 2017;6:1. 10.5455/medscience.2017.06.8687.

[8] Eltaib L, Alanazi S. Practices and attitudes concerning expiration date, unused, and expired medication disposal. Int J Med Sci Public Health. 2020;1. 10.5455/ijmsph.2020.06099202010082020.

[9] Ghosh S, LaPara TM. The effects of subtherapeutic antibiotic use in farm animals on the proliferation and persistence of antibiotic resistance among soil bacteria. ISME J. 2007;1:191–203. 10.1038/ismej.2007.31. 18043630 10.1038/ismej.2007.31

[10] Oaks JL, Gilbert M, Virani MZ, Watson RT, Meteyer CU, Rideout BA, et al. Diclofenac residues as the cause of vulture population decline in Pakistan. Nature. 2004;427:630–3. 10.1038/nature02317. 14745453 10.1038/nature02317

[11] Sanchez W, Sremski W, Piccini B, Palluel O, Maillot–Maréchal E, Betoulle S, et al. Adverse effects in wild fish living downstream from pharmaceutical manufacture discharges. Environ Int. 2011;37:1342–8. 10.1016/j.envint.2011.06.002. 21722962 10.1016/j.envint.2011.06.002

[12] Wasserfallen J–B, Bourgeois R, Büla C, Yersin B, Buclin T. Composition and Cost of Drugs Stored at Home by Elderly Patients. Ann Pharmacother. 2003;37:731–7. 10.1345/aph.1C310. 12708953 10.1345/aph.1C310

[13] Bekker CL, Gardarsdottir H, Egberts ACG, Bouvy ML, Van den Bemt BJF. Pharmacists’ Activities to Reduce Medication Waste: An International Survey. Pharmacy. 2018;6:94. 10.3390/pharmacy6030094. 30158484 10.3390/pharmacy6030094 PMC6165518

[14] Pramestutie HR, Hariadini AL, Ebtavanny TG, Illahi RK, Ilmi SN. Managing unused, damaged, and expired medications: Knowledge and attitudes among people of Malang, Indonesia. J Appl Pharm Sci. 2021. 10.7324/JAPS.2021.110912.

[15] Alfian SD, Khoiry QA, Pratama MAA, Wahyudin W, Puspitasari IM, Pradipta IS, et al. Awareness and beliefs of community pharmacists on disposal of unused and expired household medications in Indonesia: a cross–sectional study. J Pharm Health Serv Res. 2023;14:401–6. 10.1093/jphsr/rmad043.

[16] Insani WN, Qonita NA, Jannah SS, Nuraliyah NM, Supadmi W, Gatera VA, et al. Improper disposal practice of unused and expired pharmaceutical products in Indonesian households. Heliyon. 2020;6:e04551. 10.1016/j.heliyon.2020.e04551. 32760838 10.1016/j.heliyon.2020.e04551 PMC7393449

[17] Kristina SA, Wiedyaningsih C, Cahyadi A, Ridwan BA. A Survey on Medicine Disposal Practice among Households in Yogyakarta. Asian J Pharm. 2018;12:S955–8.

[18] Wulandari RD, Laksono AD, URBAN–RURAL, DISPARITY: THE UTILIZATION OF PRIMARY HEALTHCARE CENTERS AMONG ELDERLY IN EAST JAVA, INDONESIA. Indonesian J Health Adm. 2019;7:147–54. https://doi.org/10.20473/JAKI.V7I2.2019.147–154.

[19] Wasito H, Pratiwi H, Wibowo A, Solihat NK. Education and Quality Improvement of Drug Management in Family: A Case Study at Dusun Sidasari Wetan, Kubang (Edukasi dan Peningkatan Kualitas Pengelolaan Obat di Rumah Tangga: Studi Kasus di Dusun Sidasari Wetan Desa Kubangkangkung Kawunganten Cilacap). JATI EMAS (Jurnal Aplikasi Teknik dan Pengabdian Masyarakat). 2018;2:93. 10.36339/je.v2i2.160.

[20] Makki M, Hassali MA, Awaisu A, Hashmi F. The Prevalence of Unused Medications in Homes. Pharmacy. 2019;7:61. 10.3390/pharmacy7020061. 31200530 10.3390/pharmacy7020061 PMC6631141

[21] Kusuma F, Munir M, Yuda A, Hermansyah A. Assessment of medicines and potential pharmaceutical wastes management among households in Lamongan, Indonesia. Pharm Educ. 2023;23:145–8. 10.46542/pe.2023.234.145148.

[22] Nastiti A, Riyanto AR, Supriatin A, Roosmini D, Kusumah SW, Milhan R, et al. Self–Reported Pharmaceutical Storage, Use, and Improper Disposal to The Environment Among Urban Parents in Indonesia. IOP Conf Ser Earth Environ Sci. 2022;1111:012045. https://doi.org/10.1088/1755–1315/1111/1/012045.

[23] Nurdiansyah S, Asmaningrum N, Purwandari R, Ardiana A, Rosyidi K, Nur M. The Health Literacy Level among Adult Patients in Rural and Urban Public Health Centers of Pandalungan Region: A Dual–Center Comparative Study. Jurnal Kesehatan dr Soebandi. 10. 10.36858/jkds.v10i1.355

[24] Chen X, Orom H, Hay JL, Waters EA, Schofield E, Li Y, et al. Differences in Rural and Urban Health Information Access and Use. J Rural Health. 2018;35:405. 10.1111/JRH.12335. 30444935 10.1111/jrh.12335 PMC6522336

[25] Yimer A, Moges G, Kahissay MH. Household storage and disposal of unused and expired medicines in Dessie, Ethiopia: a cross–sectional study. Front Public Health. 2024;12:1422304. 10.3389/FPUBH.2024.1422304/BIBTEX. 39512718 10.3389/fpubh.2024.1422304 PMC11540675

[26] Laksono AD, Wulandari RD, Soedirham O. Urban and Rural Disparities in Hospital Utilization among Indonesian Adults. Iran J Public Health. 2019;48:247. 10.18502/ijph.v48i2.819. 31205878 PMC6556184

[27] Cuschieri S. The STROBE guidelines. Saudi J Anaesth. 2019;13:S31–4. 10.4103/sja.SJA_543_18. 30930717 10.4103/sja.SJA_543_18 PMC6398292

[28] Indonesian Ministry of Health. Laporan Survei Kesehatan Indonesia (SKI). 2023. 2023 [cited 12 Oct 2024]. Available: https://layanandata.kemkes.go.id/katalog–data/ski/ketersediaan–data/ski–2023

[29] Rogowska J, Zimmermann A. Household Pharmaceutical Waste Disposal as a Global Problem—A Review. Int J Environ Res Public Health MDPI. 2022. 10.3390/ijerph192315798. 10.3390/ijerph192315798 PMC9737308 36497873

[30] Kardas P, Agh T. Medication non–adherence as a driver of pharmaceutical waste: integrating top–down policies with bottom–up practice. Front Public Health. 2025;13. 10.3389/fpubh.2025.1714049. 10.3389/fpubh.2025.1714049 PMC12672469 41346750

[31] Srijuntrapun P, Maluangnon K. Development of guidelines for managing unused and expired medications in local communities: An engaged stakeholder waste hierarchy approach. PLoS ONE. 2026;21. 10.1371/journal.pone.0343225. 10.1371/journal.pone.0343225 PMC12965569 41790739

[32] Tabassum S, Al Samir R, Saifur TB, Tarannum N, Mamun Bhuiyan AA, Uddin MB. Unused and Expired Medicines Disposal Practices in Asian Countries. J Biosci Experimental Pharmacol. 2024;2:34–59. 10.62624/jbep00.0017.

[33] Yang C, Doshi M, Mason N. Analysis of Medications Returned During a Medication Take–Back Event. Pharmacy. 2015;3:79–88. 10.3390/pharmacy3030079. 28975905 10.3390/pharmacy3030079 PMC5597171

[34] Thach AV, Brown CM, Pope N. Consumer perceptions about a community pharmacy–based medication take back program. J Environ Manage. 2013;127:23–7. 10.1016/j.jenvman.2013.04.025. 23669605 10.1016/j.jenvman.2013.04.025

[35] Bashaar M, Thawani V, Hassali MA, Saleem F. Disposal practices of unused and expired pharmaceuticals among general public in Kabul. BMC Public Health. 2017;17:45. https://doi.org/10.1186/s12889-016-3975–z. 28061902 10.1186/s12889-016-3975-z PMC5219664

[36] Gidey MT, Birhanu AH, Tsadik AG, Welie AG, Assefa BT. Knowledge, Attitude, and Practice of Unused and Expired Medication Disposal among Patients Visiting Ayder Comprehensive Specialized Hospital. Biomed Res Int. 2020;2020:1–7. 10.1155/2020/9538127. 10.1155/2020/9538127 PMC7463377 32908927

[37] Kusturica MP, Sabo A, Tomic Z, Horvat O, Šolak Z. Storage and disposal of unused medications: knowledge, behavior, and attitudes among Serbian people. Int J Clin Pharm. 2012;34:604–10. https://doi.org/10.1007/s11096-012-9652–0. 22644600 10.1007/s11096-012-9652-0

[38] Ariffin M, Zakili TST. Household Pharmaceutical Waste Disposal in Selangor, Malaysia—Policy, Public Perception, and Current Practices. Environ Manage. 2019;64:509–19. https://doi.org/10.1007/s00267-019-01199–y. 31399770 10.1007/s00267-019-01199-y

[39] Manocha S, Suranagi UD, Sah RK, Chandane RD, Kulhare S, Goyal N, et al. Current Disposal Practices of Unused and Expired Medicines Among General Public in Delhi and National Capital Region, India. Curr Drug Saf. 2020;15:13–9. 10.2174/1574886314666191008095344. 31593533 10.2174/1574886314666191008095344

[40] AlAzmi A, AlHamdan H, Abualezz R, Bahadig F, Abonofal N, Osman M. Patients’ Knowledge and Attitude toward the Disposal of Medications. J Pharm (Cairo). 2017;2017:1–9. 10.1155/2017/8516741. 10.1155/2017/8516741 PMC5654249 29130019

[41] Massoud MA, Chami G, Al–Hindi M, Alameddine I. Assessment of Household Disposal of Pharmaceuticals in Lebanon: Management Options to Protect Water Quality and Public Health. Environ Manage. 2016;57:1125–37. https://doi.org/10.1007/s00267-016-0666–6. 26847599 10.1007/s00267-016-0666-6

[42] Inglezakis VJ, Moustakas K. Household hazardous waste management: A review. J Environ Manage. 2015;150:310–21. 10.1016/j.jenvman.2014.11.021. 25528172 10.1016/j.jenvman.2014.11.021

[43] Lubick N. Drugs in the Environment: Do Pharmaceutical Take–Back Programs Make a Difference? Environ Health Perspect. 2010;118. https://doi.org/10.1289/ehp.118–a210. 10.1289/ehp.118-a210 PMC2866706 20435558

[44] Wieczorkiewicz SM, Kassamali Z, Danziger LH. Behind Closed Doors: Medication Storage and Disposal in the Home. Ann Pharmacother. 2013;47:482–9. 10.1345/aph.1R706. 23535813 10.1345/aph.1R706

[45] Tong AYC, Peake BM, Braund R. Disposal practices for unused medications around the world. Environ Int. 2011;37:292–8. 10.1016/j.envint.2010.10.002. 20970194 10.1016/j.envint.2010.10.002

[46] Ayele Y, Mamu M. Assessment of knowledge, attitude and practice towards disposal of unused and expired pharmaceuticals among community in Harar city, Eastern Ethiopia. J Pharm Policy Pract. 2018;11:27. https://doi.org/10.1186/s40545-018-0155–9. 30459955 10.1186/s40545-018-0155-9 PMC6236888

[47] Kahsay H, Ahmedin M, Kebede B, Gebrezihar K, Araya H, Tesfay D. Assessment of Knowledge, Attitude, and Disposal Practice of Unused and Expired Pharmaceuticals in Community of Adigrat City, Northern Ethiopia. J Environ Public Health. 2020;2020:1–11. 10.1155/2020/6725423. 10.1155/2020/6725423 PMC7178471 32351582

[48] Adedeji–Adenola H, Adesina A, Obono M, Onedo T, Okafor GU, Longe M, et al. Knowledge, perception and practice of pharmaceutical waste disposal among the public in Lagos State, Nigeria. Pan Afr Med J. 2022;42. 10.11604/pamj.2022.42.106.34529. 10.11604/pamj.2022.42.106.34529 PMC9392007 36034015

[49] Vellinga A, Cormican S, Driscoll J, Furey M, O’Sullivan M, Cormican M. Public practice regarding disposal of unused medicines in Ireland. Sci Total Environ. 2014;478:98–102. 10.1016/j.scitotenv.2014.01.085. 24530589 10.1016/j.scitotenv.2014.01.085

[50] Hwang B–D. Storage and Disposal of Unused Medications for Housewives in the Busan Metropolitan City. Korean J Health Service Manage. 2013;7:69–79. 10.12811/kshsm.2013.7.2.069.

[51] Persson M, Sabelström E, Gunnarsson B. Handling of unused prescription drugs — knowledge, behaviour and attitude among Swedish people. Environ Int. 2009;35:771–4. 10.1016/j.envint.2008.10.002. 19013646 10.1016/j.envint.2008.10.002

[52] Wheeler A, Spinks J, Kelly F, Bettington E. Returning unwanted medicines to pharmacies: prescribing to reduce waste. Aust Prescr. 2018;41:78–81. 10.18773/austprescr.2018.015. 29922002 10.18773/austprescr.2018.015 PMC6003016

[53] Cyclamed C. French Drug Take Back System. 2020 [cited 14 May 2023]. Available: https://www.cyclamed.org/

[54] Alnahas F, Yeboah P, Fliedel L, Abdin AY, Alhareth K. Expired Medication: Societal, Regulatory and Ethical Aspects of a Wasted Opportunity. Int J Environ Res Public Health. 2020;17:787. 10.3390/ijerph17030787. 32012703 10.3390/ijerph17030787 PMC7037917

[55] Alfian SD, Rendrayani F, Khoiry QA, Pratama MAA, Griselda M, Pradipta IS, et al. Do pharmacists counsel customers on the disposal of unused or expired household medications? A national survey among 1,596 pharmacists in Indonesia. Saudi Pharm J. 2024;32:102020. 10.1016/j.jsps.2024.102020. 38525264 10.1016/j.jsps.2024.102020 PMC10960135

[56] Alfian SD, Adhinagoro B, Winardi DO, Angela F, Griselda M, Gathera VA, et al. Pharmacist–led interventions in addressing improper disposal practices of unused and expired household medication: A systematic review. Heliyon. 2024;10:e37764. 10.1016/j.heliyon.2024.e37764. 39315146 10.1016/j.heliyon.2024.e37764 PMC11417203

[57] Badan Pengawas Obat dan Makanan. Badan POM Canangkan Ayo Buang Sampah Obat–Gerakan Waspada Obat Ilegal. 2019 [cited 14 May 2023]. Available: https://www.pom.go.id/new/view/direct/ayo–buang–sampah–obat

[58] Alfian SD, Rendrayani F, Khoiry QA, Pratama MAA, Griselda M, Pradipta IS, et al. Do pharmacists counsel customers on the disposal of unused or expired household medications? A national survey among 1,596 pharmacists in Indonesia. Saudi Pharm J. 2024;32. 10.1016/j.jsps.2024.102020. 10.1016/j.jsps.2024.102020 PMC10960135 38525264

[59] Alfian SD, Azzahra AM, Khoiry QA, Griselda M, Puspitasari IM, Abdulah R. Pharmacists perspectives on challenges and facilitators in initiating medications take–back program in Indonesia: A qualitative study. SAGE Open Med. 2024;12. 10.1177/20503121241290968. 10.1177/20503121241290968 PMC11492182 39434985

